# Normal Hematopoietic Stem Cells within the AML Bone Marrow Have a Distinct and Higher ALDH Activity Level than Co-Existing Leukemic Stem Cells

**DOI:** 10.1371/journal.pone.0078897

**Published:** 2013-11-11

**Authors:** Gerrit J. Schuurhuis, Michael H. Meel, Floris Wouters, Lisa A. Min, Monique Terwijn, Nick A. de Jonge, Angele Kelder, Alexander N. Snel, Sonja Zweegman, Gert J. Ossenkoppele, Linda Smit

**Affiliations:** Department of Hematology, VU University Medical Center, Amsterdam, The Netherlands; Indian Institute of Toxicology Research, India

## Abstract

Persistence of leukemic stem cells (LSC) after chemotherapy is thought to be responsible for relapse and prevents the curative treatment of acute myeloid leukemia (AML) patients. LSC and normal hematopoietic stem cells (HSC) share many characteristics and co-exist in the bone marrow of AML patients. For the development of successful LSC-targeted therapy, enabling eradication of LSC while sparing HSC, the identification of differences between LSC and HSC residing within the AML bone marrow is crucial. For identification of these LSC targets, as well as for AML LSC characterization, discrimination between LSC and HSC within the AML bone marrow is imperative. Here we show that normal CD34+CD38– HSC present in AML bone marrow, identified by their lack of aberrant immunophenotypic and molecular marker expression and low scatter properties, are a distinct sub-population of cells with high ALDH activity (ALDH^bright^). The ALDH^bright^ compartment contains, besides normal HSC, more differentiated, normal CD34+CD38+ progenitors. Furthermore, we show that in CD34-negative AML, containing solely normal CD34+ cells, LSC are CD34– and ALDH^low^. In CD34-positive AML, LSC are also ALDH^low^ but can be either CD34+ or CD34–. In conclusion, although malignant AML blasts have varying ALDH activity, a common feature of all AML cases is that LSC have lower ALDH activity than the CD34+CD38– HSC that co-exist with these LSC in the AML bone marrow. Our findings form the basis for combined functionally and immunophenotypically based identification and purification of LSC and HSC within the AML bone marrow, aiming at development of highly specific anti-LSC therapy.

## Introduction

Only a small subpopulation of cells within acute myeloid leukemia (AML) is responsible for sustaining the leukemia [1]. This small subpopulation of leukemia-maintaining cells share cell surface markers with normal hematopoietic stem cells (HSC) and are capable of self-renewal and differentiation which has given them the name “leukemic stem cells” (LSC). Despite high remission rates after chemotherapy, only 30–40% of AML patients survive five years after diagnosis [2]. The main cause of present treatment failure is thought to be the insufficient eradication and survival of chemotherapy resistant LSC [3], [4]. Indeed, we have shown that high frequencies of CD34+CD38– LSC at diagnosis and after treatment predict relapse in AML [3], [5]. Beside LSC frequency, the capacity for an AML sample to give leukemic engraftment as well as recently identified LSC and HSC gene expression signatures have been linked to clinical outcome [6], [7]. Thus, LSC are prognostic and clinically important. The eradication of LSC may prevent relapse and therefore significantly improve long-term AML outcome.

LSC capable of initiating human AML in NOD/SCID mice were thought to be solely of the CD34+CD38– phenotype, similar to the normal HSC [1]. However, the LSC phenotype is more heterogeneous than initially realized and can even vary within a single AML patient [8]. Apart from the CD34+CD38– immunophenotype, other phenotypes have been associated with LSC properties such as the CD34+CD38+ and CD34– immunophenotypes [8], [9]. The CD34+CD38– cell compartment within the AML bone marrow (BM) includes both normal HSC and LSC. Selective anti-LSC therapies will be targeted to eradicate LSC while sparing HSC. Given that LSC and HSC share many features, the extent to which they differ will be a critical issue in development of LSC-targeted therapies with minimal toxicity. The search for these differences will be most relevant in HSC and LSC both obtained from the AML BM, thereby taking into account the effects of the AML microenvironment on both cell populations [10].

Discrimination and purification of CD34+CD38– LSC and CD34+CD38– HSC have been performed by using leukemia-associated proteins identified by us and others [11]–[15]. These include CLL-1, CD123 and several lineage markers [12]–[14]. CLL-1 is expressed on part of normal progenitors and in a portion of AML cases on LSC, but is absent on HSC [13]. Also lineage markers are absent on HSC, while in part of the AML cases, are expressed on both leukemic stem and progenitor cells [11], [12]. In general, immunophenotypic leukemia-associated markers are not expressed on all leukemic cells and not in all AML patients resulting in an inability to use a single marker for LSC identification in the whole AML patient population. This emphasizes the need for identification of additional markers of malignancy to detect LSC and discriminate these from HSC in all AML patients.

Recently, we discovered additional differences between HSC and LSC in both AML and chronic myeloid leukemia (CML) [5], [16]. We showed that normal and malignant stem cells can be identified by differences in light scatter properties (forward scatter, FSC and side scatter, SSC) and CD34 and/or CD45 expression. HSC values were 1–1.4 times (FSC) and 1–1.7 times (SSC) that of lymphocytes and defined as FSC/SSC^low^. LSC values are more than 1.4 times (FSC) and 1.7 times (SSC) that of the lymphocytes and defined as FSC/SSC^high^. However, still in a considerable part of AML cases neither marker expression nor the combination with scatter properties is able to distinguish LSC from HSC. Therefore, we searched for a functional difference between normal HSC and LSC within the AML BM.

Aldehyde dehydrogenases (ALDHs) are cytosolic enzymes involved in the conversion of retinol (Vitamin A) to retinoic acids and important in HSC maturation, differentiation and loss of quiescence [17], [18]. These enzymes play a major role in the protection of BM progenitors from the cytotoxic effects of cyclophosphamide [19], [20] and increased the resistance to various other cytotoxic agents [20], [21]. The elevated expression of ALDH has been demonstrated in progenitors as compared to other hematopoietic cells like lymphocytes. Hematopoietic cells with high ALDH activity were enriched in the CD34+Lin- compartment indicating that ALDH activity is a marker for primitive hematopoietic stem/progenitor cells [22]–[27]. In AML, ALDH activity in the blast cells have been shown to correlate with clinical outcome [28], [29].

We investigated the ALDH activity in LSC as compared to normal CD34+CD38– HSC, both present in the BM of AML patients. We used discriminative aberrant cell surface markers and scatter aberrancies for the identification of HSC and LSC and analysed AML samples both from CD34-positive and CD34-negative AML. In this paper, we have demonstrated that, in both AML subtypes, high ALDH activity is an unique marker distinguishing CD34+CD38– HSC from CD34+ and CD34– LSC present within the BM of all the AML cases studied. In CD34-negative AML, CD34+CD38– stem cells are all normal (HSC) and have high ALDH activity. The LSC within this AML subtype are ALDH^low^ and of the CD34– immunophenotype. In CD34-positive AML, CD34+CD38– HSC are, like in CD34-negative AML, ALDH^bright^ while the LSC are ALDH^low^ and either of the CD34+ or CD34– immunophenotype. This marked difference in ALDH activity between HSC and LSC offers an opportunity for identification and purification of LSC and CD34+CD38– HSC in every AML case.

## Results

### AML can be Divided in Two Subgroups Based on the Frequency and Pattern of CD34 Expression

**Figure 1:**
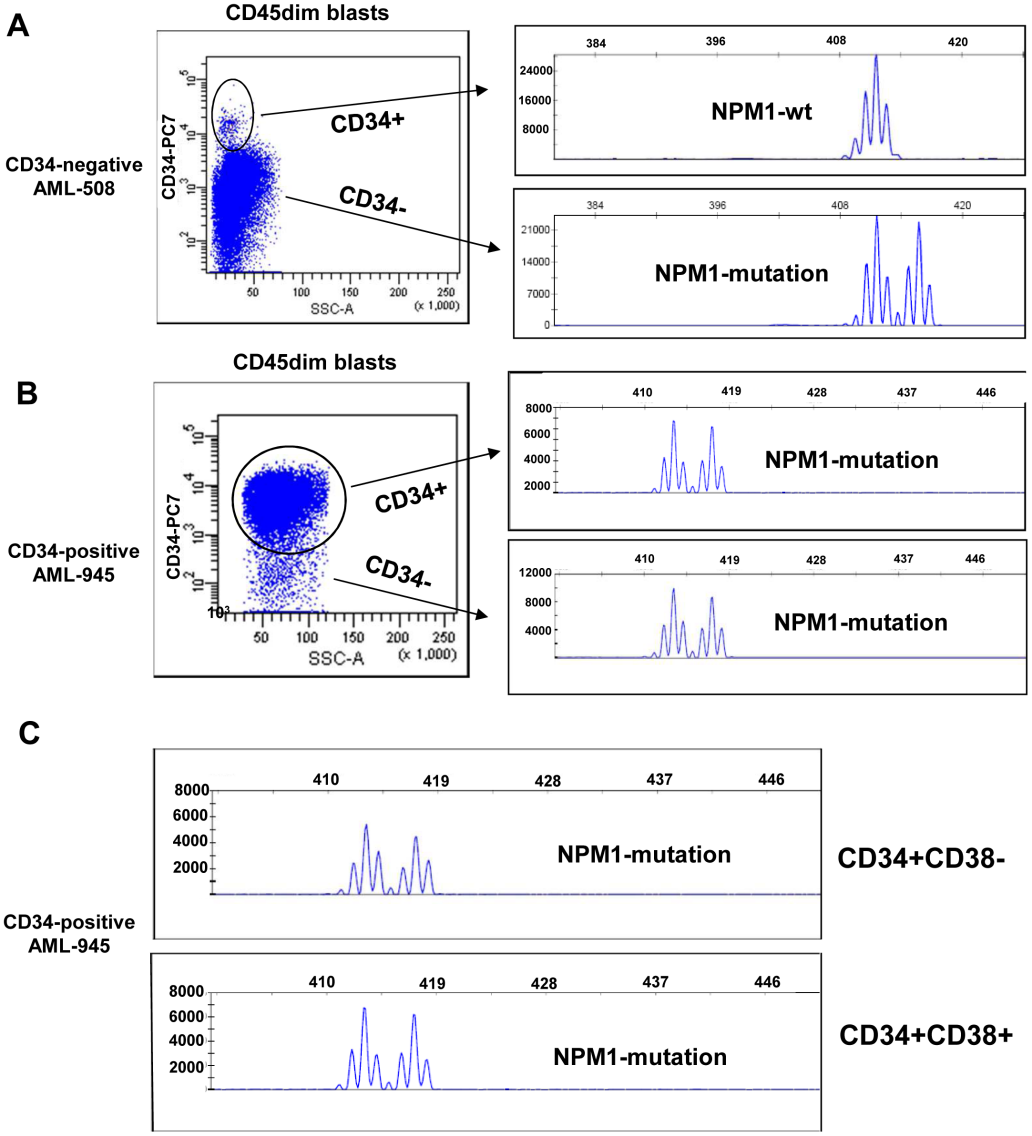
AML can be divided in two subtypes based on frequency and pattern of CD34 expression. Representative flow cytometric staining patterns of CD34 expression (CD34 versus SCC) are shown for (**A**) a CD34-negative AML case (0.03% CD34+ cells within the blast compartment) and (**B and C**) a CD34-positive AML case (72% CD34+ cells within the blast compartment). The CD34-negative AML cases contain a discrete, usually less than 1%, CD34+ cell population. This CD34+ population is completely devoid of molecular aberrancies (in this case mutated NPM1)(**A**). The CD34-positive AML case contains a large CD34+ cell population, usually more than 1%, which contains the leukemia-associated mutated NPM1 protein (**B**). The CD34+CD38– and CD34+CD38– fractions from a CD34-positive case contain the FLT3-ITD and NPM1 mutation (**C**).

We have defined two groups of AML cases based on frequency and pattern of CD34 expression in the blast compartment. One of the subtypes, which we call CD34-negative AML, has a small, usually less than 1%, population of CD34+ cells which are cytogenetically and molecularly normal, as assessed by FISH analyses [30] and PCR (Figure 1A, AML-508). Thus, CD34-negative AML lacks CD34+ leukemic cells and consequently lacks also CD34+CD38– LSC. LSC in CD34-negative AML cases are of the CD34– immunophenotype. CD34-positive AML cases contain both leukemic CD34+ and CD34– cells, shown by the presence of molecular aberrancies in the CD34+ as well as the CD34– cell compartments (Figure 1B, AML-945), Within the CD34+ compartment, CD34+CD38– and CD34+CD38+ fractions contain mutated NPM1. Thus, LSC within CD34-positive AML can be either CD34+ or CD34– [8].

### In Healthy BM, CD34+CD38− HSC are ALDHbright while ALDH Activity Decreases upon Differentiation

**Figure 2:**
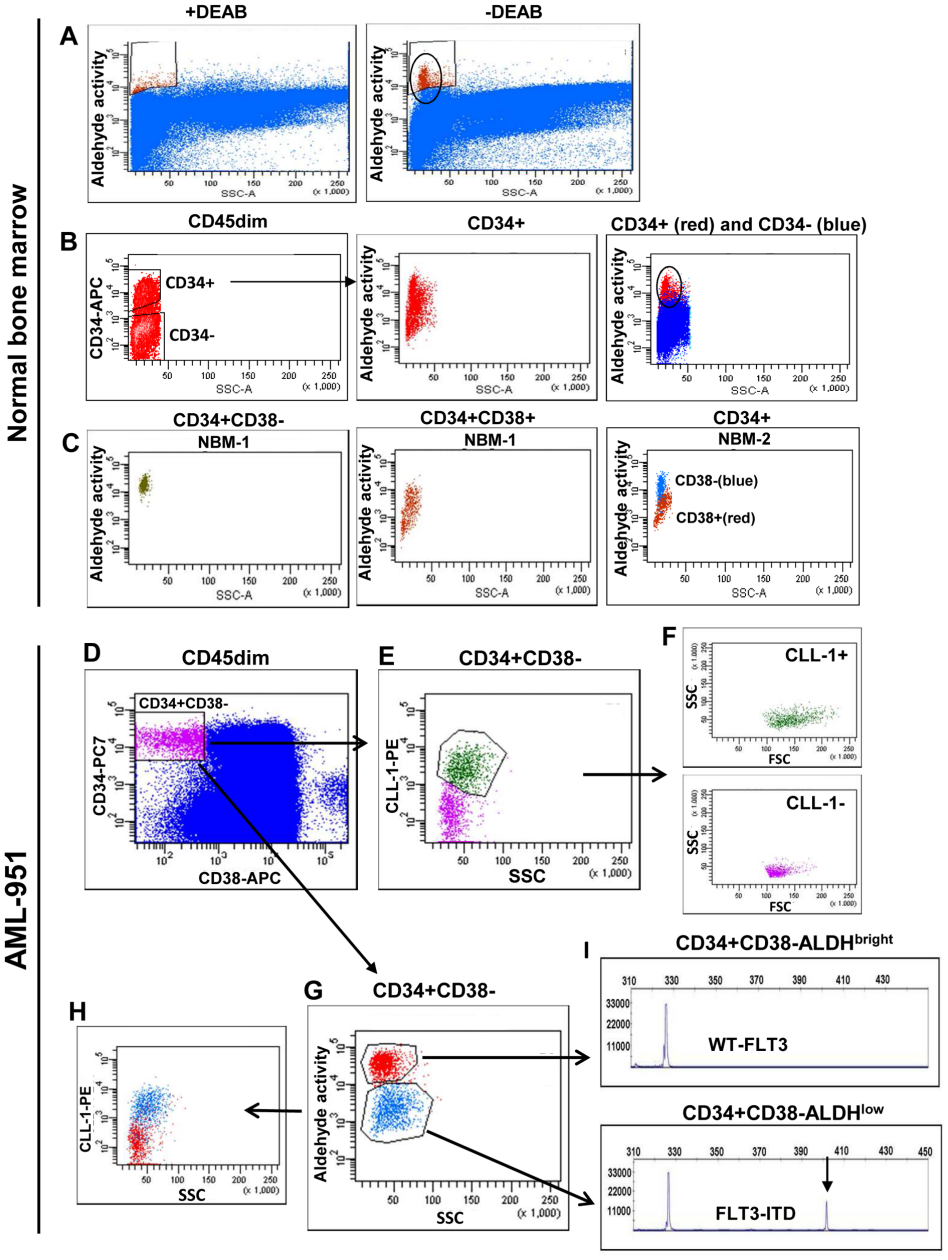
CD34+CD38– HSC have higher ALDH activity than co-existing CD34+CD38– LSC. (**A–C**) CD34+CD38– HSC within normal bone marrow are ALDH^bright^ and the level of ALDH activity decreases upon differentiation to CD38+ progenitors. Representative flow cytometric ALDH activity patterns (ALDH versus SSC) are shown for (**A**) total CD45dim normal bone marrow cells treated with or without DEAB and (**B**) CD45dimCD34+ cells (middle panel) and both CD45dimCD34+ cells (red) and CD45dimCD34– cells (blue)(right panel). In C, the ALDH activity versus SCC of CD34+CD38– stem cells (panel 1 in green and panel 3 in bleu) and CD34+CD38+ progenitor cells (red in panel 2 and panel 3) from the normal BM is shown. (**D–I**) In CD34-positive AML, the ALDH activity of CD34+CD38– HSC is higher than that of the CD34+CD38– LSC. The aldefluor assay was performed on cells of a CD34-positive AML case (AML-951) and cells were subsequently labeled with anti-CD45 PERCP, anti-CD34 PC7, anti-CD38 APC and anti-CLL1 PE. The CD34+CD38– stem cells (**D**, purple) showed to be partly CLL-1+ and partly CLL-1– (**E**). The CLL-1+ stem cells (green) are FSC/SSC^high^ as compared to the CLL-1– cells (purple)(**F**). Investigation of the ALDH activity of the CD34+CD38– compartment showed that the CD34+CD38– stem cell population (**D**) segregates into an ALDH^bright^ (red) and an ALDH^low^ (blue) population (**G**). The ALDH^bright^ cells (red) are CLL-1 negative (H) and contain only wild type FLT3 kinase (**I,** upper panel). The ALDH^low^ cells (blue) are largely CLL-1 positive (**H**) and contain FLT3-ITD+ cells (**I**, lower panel). The arrow indicates the FLT3-ITD.

BM from a healthy donor contains a small population of cells with high ALDH activity (Figure 2A). This ALDH activity can be inhibited by incubation with diethylaminobenzaldehyde (DEAB), an inhibitor of the enzymatic activity of the ALDH proteins (Figure 2A). The CD34+ population within normal BM contained this same population of high ALDH activity cells (Figure 2B, circle), which we called ALDH^bright^. The CD34– cell population within normal BM is ALDH^low^ (Figure 2B, right panel, blue). The stem cell compartment within normal BM, defined as the CD34+CD38– cells, was completely retained in this high ALDH activity compartment (two examples in Figure 2C, left panel, green and right panel, blue) and visible as a single population, while the CD34+CD38+ more differentiated progenitor cells were ALDH^low^ (Figure 2C, middle and right panels, red). Thus, ALDH activity is high in CD34+CD38– HSC and decreases when cells become more differentiated. The normal BM CD34+CD38–ALDH^bright^ cells comprised the complete (>99%) CD34+CD38– population of cells (n = 5).

### In CD34-positive AML, the CD34+CD38− ALDHbright Cells Retain the HSC while CD34+CD38− ALDHlow Cells are LSC

In the CD34+CD38– stem cell compartment of CD34-positive AML cases (n = 19) two separate ALDH activity populations, which we refer to as ALDH^bright^ and ALDH^low^ (Table 1, Figure 2D and 2G), were seen. The CD34+CD38– ALDH^low^ compartment was not observed in normal donor BM as outlined in the left panel of Figure 2C. In CD34-positive AML cases, CD34+CD38– cells that express aberrant leukemia-associated immunophenotypic markers are neoplastic [12], [31]. Expression of these leukemia-associated markers is often not on all leukemic cells and consequently not on all LSC. Therefore, CD34+CD38– cells that lack aberrant marker expression can be either HSC or LSC. In the marker-negative CD34+CD38– population we can, in many cases, discriminate between HSC and LSC using additional characteristics, such as scatter properties. HSC have a lower FSC and/or SSC than the LSC population [5], [16]. On the basis of expression of aberrant markers and scatter properties we showed that CD34+CD38– ALDH^bright^ cells in CD34-positive AML (n = 19) had no expression of immunophenotypic leukemia-associated markers (median 0.03% marker expression on CD34+CD38– ALDH^bright^ cells, Table 1) and are lower in FSC and/or SSC than ALDH^low^ cells (data not shown), strongly suggesting that ALDH^bright^ cells are CD34+CD38– HSC. The level of ALDH activity corresponds with that of normal BM CD34+CD38– HSC (Figure 2C, right and middle panel). The ALDH^low^ cells had aberrant marker expression (frequency corresponds with that of marker expression on leukemic CD45dim blasts of the corresponding AML, median is 25% marker expression on CD34+CD38– ALDH^low^ cells, Table 1) and a higher FSC/SSC, suggesting to be CD34+CD38– LSC. Thus, putative normal HSC are contained within the ALDH^bright^ compartment while LSC are ALDH^low^.

**Table 1:** CD34+CD38–stem cell frequencies and their marker expression in the ALDH^bright^ and ALDH^low^ compartment in CD34-positive AML.

CD34-pos AML *de novo*	Marker	% CD34+CD38– ALDH^bright^	Marker+ %& #
575	CD33	51.3	1.7 (2c) ‡
670	CLL-1	54.4	0.3 (1c)
726	CD7	34.5	0.3 (3c)
808	none	22.7	–
945	CLL-1/CD19	44.6	1.1 (2c)
951	CLL-1	51.9	1.5 (23c)
966	CD7	58.9	–
1013	CLL-1	22	0.67 (15c)
1016	CD11b	0.25	0
1022	CLL-1	38	0
1030	CD22	10	3 (2c)
1034	CLL-1	3	0
1036	CLL-1	20	0
1047	CD7	5.5	0
1048	CD7	92	0.03
1057	CD19	7	0
1263	CD33/CD123	84	0
1305	CLL-1	58	3.5 (6c)
1320	CD2	92.2	0
*Median*		*38*	*0.03*
**CD34-pos AML ** ***de novo***	**Marker**	**% CD34+CD38**– **ALDH^low^**	**Marker+ %** & #
575	CD33	0.01	100
670	CLL-1	0.1	4.5
726	CD7	0	–
808	none	0.2	–
945	CLL-1/CD19	0.1	86.4
951	CLL-1	0.1	76.7
966	CD7	0.5	24.4
1013	CLL-1	2.36	15
1016	CD11b	0.28	13
1022	CLL-1	0	–
1030	CD22	3.5	6
1034	CLL-1	0.12	40
1036	CLL-1	15	25
1047	CD7	0.18	12
1048	CD7	34	12
1057	CD19	0.15	67
1263	CD33/CD123	1.7	99
1305	CLL-1	0.7	84
1320	CD2	0.6	7.2
*Median*		*0.2*	*24.7*

Detection of immunophenotypic aberrancies in CD34+CD38– ALDH^bright^ and ALDH^low^ compartments of CD34-positive AML.

* The percentage of CD34+CD38– cells in the total ALDH^bright^ or ALDH^low^ compartments is indicated.

& in the ALDH^bright^ CD34+CD38– compartment the aberrant marker expression, both CLL-1 and lineage markers, is indicative for the neoplastic character of the cells.

# Percentage expression does not quantitatively correlate with % of neoplastic cells: a shift in fluorescence of a total cell population may result e.g. in 50% marker expression based on normalization by isotype controls of negative cell populations.

‡ data in between parenthesis represent number of cells in cases where there is <25 cells positive for a marker.

This putative normal HSC, CD34+CD38– ALDH^bright^, cell population of 7 of these 19 CD34-positive AML cases was essentially devoid of cells with the leukemia-specific cytogenetic abnormalities FLT3-ITD and/or mutated NPM1 (Table 2). An ALDH activity analysis of such a CD34-positive AML, in this case FLT3-ITD-positive, is shown in Figure 2D–I (AML-951). The CD34+CD38– compartment contains both CLL-1+ and CLL-1– cells (Figure 2E), whereby the CLL-1+ stem cells are in general FSC/SSC^high^ as compared to the CLL-1– cells (Figure 2F). Analysis of the ALDH activity of the CD34+CD38– compartment showed that it segregates into an ALDH^bright^ and an ALDH^low^ population (Figure 2G). The ALDH^bright^ cells are CLL-1– (Figure 2H), FSC/SSC^low^ (Figure 2F) and contained only wild type FLT3 kinase (Figure 2I, upper panel), indicating HSC. The ALDH^low^ cells are for a major part CLL-1+ (Figure 2H), FSC/SSC^high^ (Figure 2F) and contain FLT3-ITD+ cells (Figure 2I, lower panel), indicating LSC.

**Table 2:** Presence of FLT3-ITD and mutated NPM1 in ALDH^bright^ and ALDH^low^ compartments in CD34-positive AML.

Patient # FLT3-ITD	CD34– cells	CD34+CD38 ALDH^low^	CD34+CD38– ALDH^bright^
575	pos	50% ITD	wt
808	pos	50% ITD	wt
951	pos	42% ITD	wt
966	pos	43% ITD	wt
1263	pos	60% ITD	wt
**Patient # NPM1**	**CD34**– **cells**	**CD34+CD38 ALDH^low^**	**CD34+CD38 ALDH^bright^**
575	mut	mut	wt
808	mut	mut	wt
945	mut	mut	wt (<5%)
670	mut	mut	wt

Detection of molecular aberrancies in CD34+CD38– ALDH^bright^ and ALDH^low^ compartments of CD34-positive AML. The FLT3-ITD percentage is determined in the total leukemic blast population (data not shown), the CD34– cell population and the CD34+CD38-ALDH^low^ and CD34+CD38-ALDH^bright^ compartments. The NPM1 mutation analysis is not quantative. # the ALDH^bright^ compartment contained in one case a small mutant peak (AML-945), likely caused by relatively poor separation of ALDH^bright^ and ALDH^low^ populations due to overlap in the boundary region.

**Figure 3:**
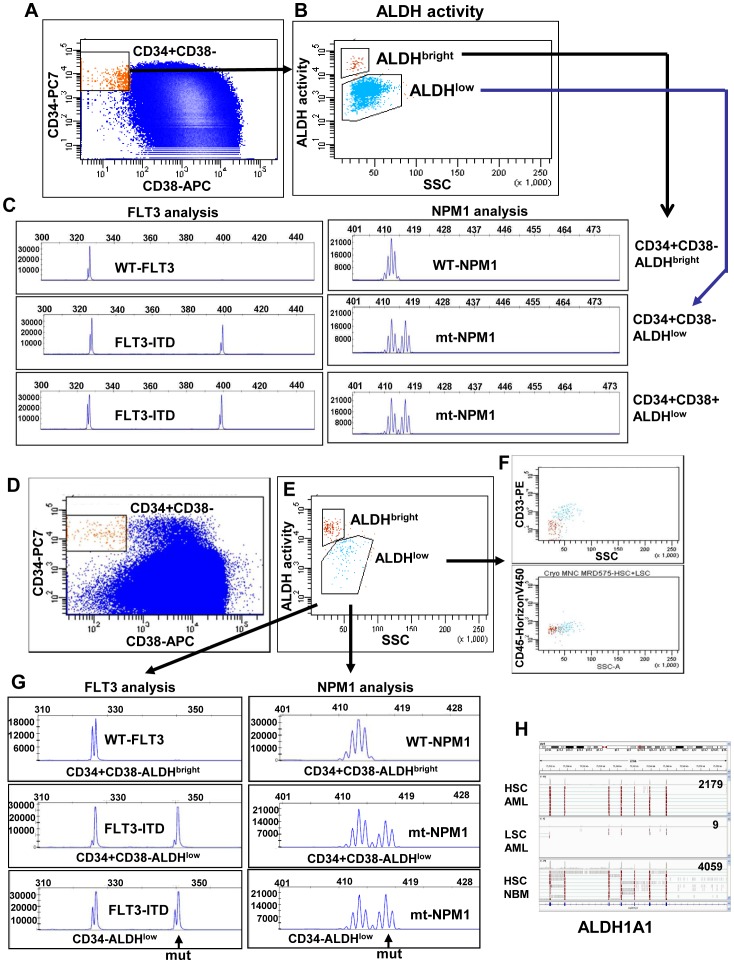
ALDH^bright^ CD34+CD38– cells are devoid of both the FLT3 and NPM1 mutation. AML-808 contains both FLT3-ITD and mutated NPM1 and has no immunophenotypic aberrancy present. However, AML-808 can be segregated into a normal and a leukemic CD34+CD38– compartment by measuring ALDH activity (**A–C**). The CD34+CD38– stem cells (**A**, orange) segregate into two separate ALDH compartments (**B**). The CD34+CD38–ALDH^bright^ cells of AML-808 do neither contain FLT3-ITD nor mutated NPM1 (**C**, upper panels), while the CD34+CD38-ALDH^low^ cells (blue) do (**C**, middle panel). The ALDH^low^ cells have both mutations (**C**, lower panel). AML-575 contains both FLT3-ITD and mutated NPM1. The CD34+CD38– cells (red) segregate into two separate ALDH compartments (**D**, middle panel). The ALDH^bright^ (red) cells within AML-575 lack the immunophenotypic aberrancy CD33 and are low in SSC compared to the ALDH^low^ cells (blue)(**F**). The CD34+CD38-ALDH^bright^ cells do neither contain FLT3-ITD nor mutated NPM1 (**G**, upper panels), while the CD34+CD38-ALDH^low^ cells do (**G**, middle panel). The ALDH^low^ cells have both mutations (**G**, lower panel). (**H**) CD34+CD38– HSC, CD34+CD38– LSC (purification based on CLL1+, CD34 expression and scatter properties) from AML-598, and CD34+CD38– HSC from normal BM were purified. RNA was isolated and RNA sequencing was performed. The most abundant ALDH mRNA expressed within both normal HSC fractions (AML and normal BM) was the ALDH1A1 enzyme. LSC had hardly any expression of the ALDH1A1 form. Numbers indicate the amount of ALDH1A1 reads within that fraction.

Development of acute leukemia follows the rules of the two-hit model; cells have to acquire a mutation interfering with differentiation and a mutation conferring a proliferative advantage to become neoplastic [32]. Consequently, there is a possibility that hematopoietic cells with only one detectable aberrant leukemia-associated molecular mutation still have a normal phenotype. To confirm that the ALDH^bright^ CD34+CD38– cells are normal HSC and the ALDH^low^ CD34+CD38– cells are LSC we analysed the presence of molecular aberrancies in the ALDH compartments from CD34-positive AML cases with two molecular aberrancies, FLT3-ITD and mutated NPM1 (AML-575 and AML-808). CD34+CD38– AML cells can be divided in an ALDH^bright^ and ALDH^low^ compartment (Figure 3A,B, AML-808 and 3D,E, AML-575). In case of AML 575, the ALDH^bright^ cells are negative for CD33 and low in SSC strongly suggesting normal HSC (Figure 3F). In both these AML cases the ALDH^bright^ compartment contains neither an FLT3-ITD nor an NPM1 mutation (Figure 3C, AML-808 and 3G, AML-575 upper panels), indicating HSC, while the ALDH^low^ compartment has both these leukemia-associated mutations (Figure 3C, AML-808 and 3G, AML-575 middle panels), indicating LSC.

### In the Absence of Immunophenotypic Marker Aberrancies, CD34+CD38− HSC can be Discriminated from CD34+CD38− LSC Solely on ALDH Activity

In part of the AML cases (1/19 in our CD34-positive AML population, AML-808), the CD34+CD38– compartment can not be segregated into HSC and LSC by expression of aberrant immunophenotypic markers or by scatter properties. In such cases, nevertheless ALDH activity identified two separate populations within the CD34+CD38– compartment (Figure 3A and 3B). The ALDH^bright^ CD34+CD38– cells lacked both FLT3-ITD and mutated NPM1 (Figure 3C, upper panels), indicating normal HSC while the ALDH^low^ CD34+CD38– cells contained the FLT3-ITD and mutated NPM1 (Figure 3C, lower panels), indicating LSC. Thus, even in the absence of marker aberrancies, CD34+CD38– HSC can be discriminated from CD34+CD38– LSC based on ALDH activity.

Different isoforms of ALDH might contribute to the ALDH activity profile seen. To search for the ALDH enzyme(s) responsible for the high ALDH activity seen in HSC as compared to LSC, the HSC from healthy donor BM and CD34+CD38– HSC and LSC from the AML BM were purified. HSC and LSC purified from the AML BM were defined as CLL-1-, FSC/SSC^low^ and CLL-1+, FSC/SSC^high.,^ respectively. These stem cell fractions were analysed for ALDH isoform expression by RNA-sequencing. The putative HSC contained within the AML BM have high expression of the ALDH1A1 enzyme as compared to almost no expression in the LSC (Figure 3H, AML-598). Moreover, in HSC from the BM of a healthy donor, like in HSC from the AML BM, the ALDH1A1 isoform is highly expressed (Figure 3H). In HSC and LSC within this AML BM other ALDH enzymes are expressed be it at much lower level than ALDH1A1 in the HSC. ALDH3B1 is the only ALDH member that is higher expressed in LSC as compared to HSC (5 fold).

### CD34-positive AML: Inter-Individual Variation in the Difference in ALDH Activity between LSC and HSC

With flow cytometry analysis, the relative ALDH activity can be measured as mean fluorescence intensity (MFI) level of the population. We standardized the ALDH-MFI values of normal and neoplastic stem cell candidates by dividing these by the ALDH-MFI value of lymphocytes present within the same sample. Lymphocytes are negative for ALDH, resulting in MFI values that can be considered as background signal and as a stable characteristic of the individual sample. Treatment with DEAB was used in 9/19 samples as an extra control and showed similar basement ALDH activity levels in most samples used (Table S1). From these 9 CD34-positive AML cases the CD34+CD38– HSC and CD34+CD38– LSC, as defined by marker expression and scatter properties, were analysed for their ALDH activity MFI. The marker-negative compartment (containing HSC) had median 6.9-fold (range 1.7–28.9) higher ALDH activity than the marker-positive LSC compartment (Table S1). This way of calculation showed marked variations in the fold difference in ALDH activity of HSC as compared to LSC in individual samples. There are cases where LSC ALDH activity levels come close to levels in HSC (Figure S1, AML-1048) while in others, LSC and HSC ALDH activity levels are far apart (Figure S1, AML-1036), indicating inter-individual variation in the difference between ALDH activity in LSC and HSC. Inhibition of the high activity of ALDH^bright^ CD34+CD38– cells by DEAB was not in all cases till the same level as the LSC present in the same sample (Figure S1, AML-1036 and AML-1013). The ALDH^low^ CD34+CD38– cell compartment segregated, in some AML cases, in a population which could be inhibited by DEAB and a population which could not (Figure S1, AML-1048), suggesting the existence of LSC populations with various levels of ALDH activity within one CD34+CD38– ALDH^low^ compartment. Some AML cases had mainly normal ALDH^bright^ CD34+CD38– cells within the BM (Figure S1, AML-1013).

### In CD34-negative AML, CD34+CD38– Cells have High ALDH Activity and are Normal while LSC are CD34– and ALDHlow

**Figure 4:**
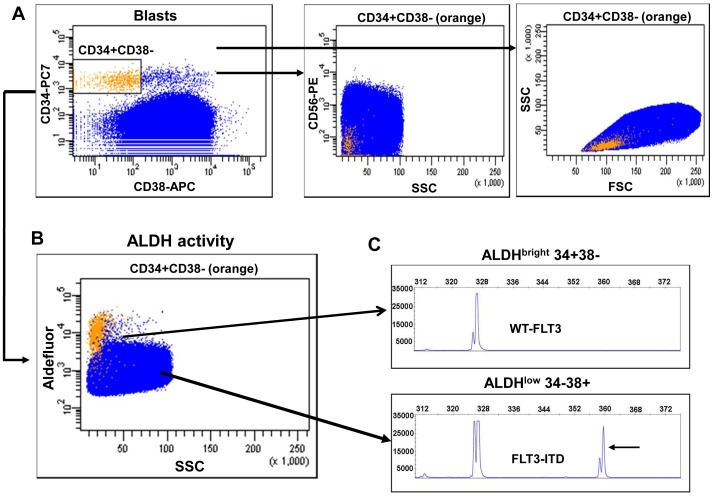
The ALDH activity of CD34+CD38– HSC is higher than the CD34– bulk in CD34-negative AML. The aldefluor assay was performed on the bulk of cells (AML-464). Cells were subsequently labeled with anti-CD45 PERCP, anti-CD34 PC7, anti-CD38 APC and anti-CD56 PE. (**A**) The CD34+CD38– stem cells (**A**, left panel, orange) were negative for CD56 (**A**, middle panel, CD34+CD38– in orange) and low in FSC/SCC (**A**, right panel). (**B**) The ALDH activity of these CD34+CD38– cells is high and shows a separate SCC^low^ALDH^bright^ population of cells (**B**, CD34+CD38– cells in orange). (**C**) Detection of FLT3-ITD by length fragment analysis showed that the ALDH^bright^ compartment was devoid of FLT3-ITD (top) while the ALDH^low^ compartment has the FLT-ITD (bottom); arrow indicate the mutation.

In CD34-negative AML, all the CD34+CD38– cells have a normal phenotype and consequently LSC are CD34– (Figure 1A) [30]. In all CD34-negative AML cases that we analysed (n = 13), the CD34+CD38– cells were retained in the ALDH^bright^ compartment while there were essentially no CD34+CD38– cells in the ALDH^low^ compartment (Table 3). The few CD34+CD38– cells that were ALDH^low^ meet the criteria FSC/SSC^low^, confirming the absence of CD34+ LSC in CD34-negative AML cases (Table 3, median 48% CD34+CD38– in ALDH^bright^ and median 0% CD34+CD38– in ALDH^low^). The bulk of the AML (CD34– cells) were ALDH^low^ (Table 3, example in Figure 4A and B).

**Table 3:** CD34+CD38– frequency and aberrant marker expression in ALDH^bright^ and ALDH^low^ compartments in CD34-negative AML.

CD34-neg AML *de novo*	Marker	% CD34+CD38− ALDH^bright^ *	Marker+ % &
464	CD56	83.8	0
508	CLL-1	35	0
813	CLL-1	24.6	0.1 (2c) ‡
822	CLL-1	50.4	0
1021	CLL-1	75	0
1024	CD7	9	0
1027	CLL-1	48	–
1028	CD7	60	0
1035	CLL-1	70	0
1045	CLL-1	14	0
1054	CD56	3	0
1063	CLL-1	57	0
1276	CLL-1	7.9	0.1
*Median*		*48*	*0*
**CD34-neg AML ** ***de novo***	**Marker**	**% CD34+CD38− ALDH^low^** *	**Marker+ %** &
464	CD56	0	–
508	CLL-1	0	–
813	CLL-1	0	–
822	CLL-1	0	–
1021	CLL-1	0.89	24
1024	CD7	2.7 (10c)	0
1027	CLL-1	1.9 (5c)	–
1028	CD7	0.05	0.44 (1c)
1035	CLL-1	0.02 (14c)	20 (9c)
1045	CLL-1	0	–
1054	CD56	0	–
1063	CLL-1	0.01 (8c)	–
1276	CLL-1	0	–
*Median*		*0*	*10.22*

Detection of immunophenotypic aberrancies in CD34+CD38– ALDH^bright^ and ALDH^low^ compartments of CD34-negative AML.

* Percentage of CD34+CD38– cells within the ALDH^bright^ or ALDH^low^ compartments.

& in the ALDH^bright^ CD34+CD38– compartment, the aberrant marker expression, both CLL-1 and lineage markers, is indicative for the neoplastic character of the cells.

‡ data in between parenthesis represent number of cells in cases where there is <25 cells with a marker. The ALDH^low^ compartment in CD34-negative AML cases is essentially devoid of CD34+CD38– stem cells.

In CD34-negative AML cases, ALDH^bright^ cells lack expression of aberrant immunophenotypic markers (Table 3, median 0%), have low scatter properties (not shown) but most importantly are devoid of the molecular aberrancies FLT3-ITD and mutated NPM1 (Table 4, example Figure 4C AML-464). In AML-464, ALDH^bright^ CD34+CD38– cells lack the aberrant marker CD56 present in the leukemic blasts of this patient (Figure 4A **,** middle panel) and have low FSC/SSC (Figure 4A, right panel). The ALDH^bright^ cells are devoid of FLT3-ITD (Figure 4C, upper panel) and the NPM1 mutation (data not shown) while the ALDH^low^ CD34– compartment contained FLT3-ITD positive cells (Figure 4C lower panel) and mutated NPM1 (not shown).

**Table 4:** The presence of FLT3-ITD and mutated NPM1 in ALDH^bright^ and ALDH^low^ compartments in CD34-negative AML.

Patient # FLT3-ITD	CD34− ALDH^low^	CD34+CD38− ALDH^bright^
1027	48% ITD	0% ITD
464	32% ITD	0% ITD
**Patient # NPM1**	**CD34− ALDH^low^**	**CD34+CD38 ALDH^bright^**
822	mut	wt
1276	mut	wt
464	mut	wt
508	mut	wt

Detection of molecular aberrancies in CD34+CD38– ALDH^bright^ and ALDH^low^ compartments of CD34-negative AML. The molecular nature of the CD34+CD38-ALDH^bright^ and CD34– compartments in CD34-negative AML cases. Detection of FLT3-ITD and mutated NPM1 was performed in the various cell fractions. The FLT3-ITD percentage is determined in the total leukemic blast population (data not shown), the CD34+CD38-ALDH^bright^ and CD34– compartments. The NPM1 mutation analysis is not quantative.

We have studied the presence of molecular aberrancies in the ALDH compartments in five CD34-negative AML cases (Table 4). The ALDH^bright^ CD34+CD38– compartment is devoid of molecular mutations (Table 4) in all the five AML patients. The ALDH^low^ compartment contained almost exclusively CD34– cells having aberrant marker expression and molecular mutations (Table 3 and 4, Figure 4).

### CD34-negative AML: Inter-individual Variation in the Difference in ALDH Activity between CD34+CD38-HSC and CD34– cells

In CD34-negative AML, we standardized the ALDH-MFI values of normal stem cell candidates and CD34– neoplastic cells by dividing these by the ALDH-MFI value of lymphocytes present within the same AML sample (Table S2). Eight CD34-negative AML cases were treated with and without DEAB and in these we analysed the ALDH activity MFI level of the CD34+CD38– HSC, defined by lack of immunophenotypic marker expression, and the ALDH activity MFI level of the CD34– cells. The marker negative CD34+CD38– compartment (HSC) had a median 4.3-fold (range 2.2–18.7) higher ALDH activity than the CD34– cells (Table S2).

### The ALDHbright Compartment in Both CD34-positive and CD34-negative AML Contained CD34+CD38+ Progenitors with a Normal Phenotype

**Figure 5:**
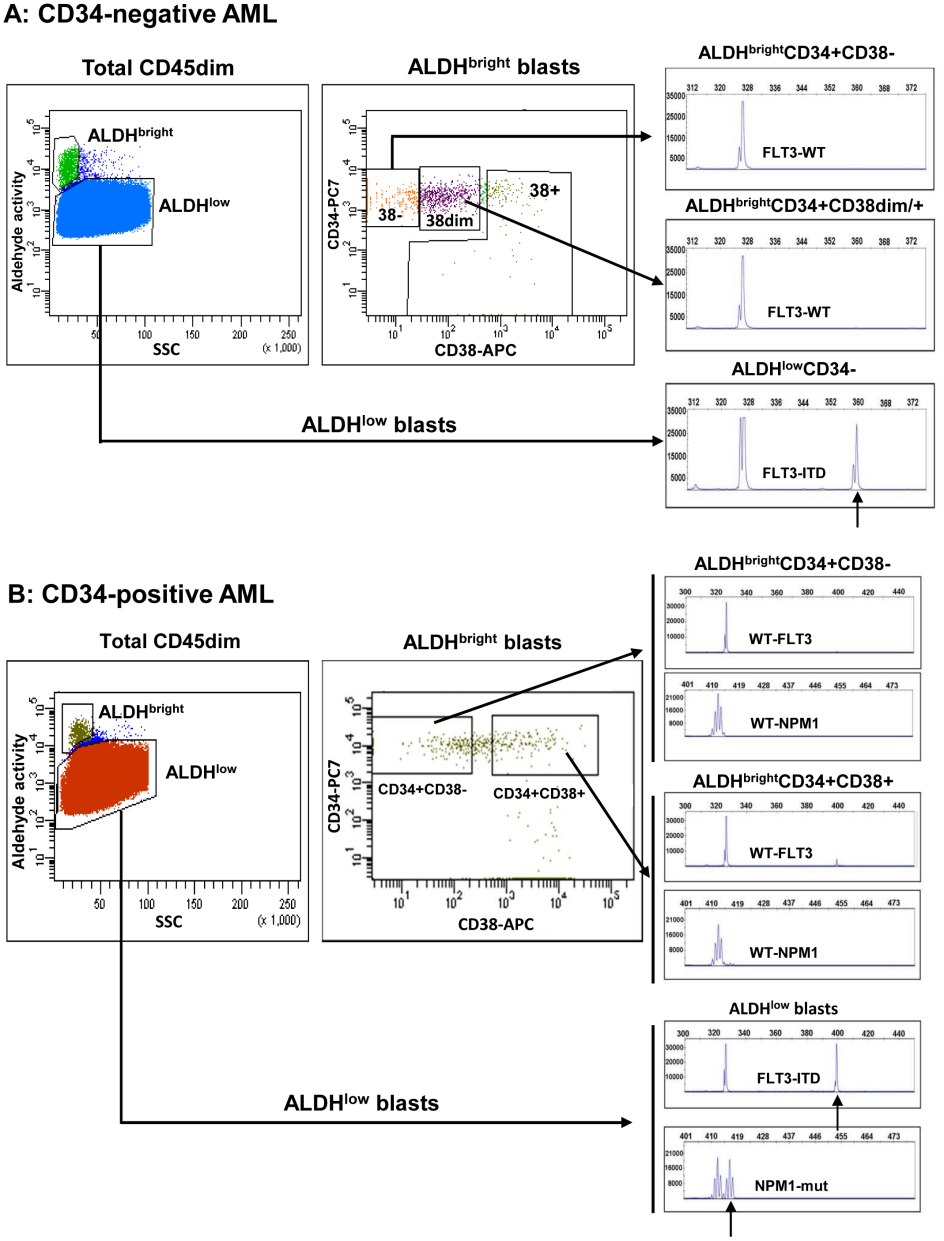
The presence of FLT3-ITD and mutated NPM1 in ALDH^bright^ CD34+CD38− progenitor populations. (**A**) In CD34-negative AML cases, the ALDH^bright^ population of AML cells consists, besides CD34+CD38− stem cells of CD38dim and CD38+ progenitors (**A**, middle panel, AML-464) (Table S3). The complete ALDH^bright^ compartment in CD34-negative AML has a normal phenotype as proven by the absence of FLT3-ITD in the CD34+CD38− (**A**, right upper panel) as well as the CD38dim (**A**, right middle panel) and CD38+ (data not shown) population of cells. The ALDH^low^ compartment contains the FLT3-ITD (arrow in right lower panel), indicating leukemic cells. (**B**) In CD34-positive AML cases, the ALDH^bright^ population contains, apart from normal CD34+CD38− HSC, CD34+CD38+ progenitors and CD34− cells (Figure 5B middle panel)(Table S3). The ALDH^bright^ CD34+CD38− stem cells lack molecular aberrancies (**B**, right upper two panels) and are therefore normal. The CD34+CD38+ progenitor compartment (**B,** right middle two panels) in this case has very small FLT3-ITD and NPM1 mutant peaks likely originating from purification of ALDH^low^ cells within the ALDH^bright^ compartment. The ALDH^low^ compartment is largely neoplastic (**B**, right lower 2 panels).

The ALDH^bright^ compartment in the BM of both CD34-positive and CD34-negative AML cases contained CD34+CD38– HSC, more differentiated CD34+CD38+ progenitors and CD34– cells (Figure 5A and 5B, Table S3). The CD34+CD38+ ALDH^bright^ cells in CD34-negative AML cases neither expressed an aberrant lineage marker, nor had a molecular aberrancy as tested for four CD34-negative AML cases (Table S3A, Figure 5A, AML-464). In CD34-positive AML, the CD34+CD38+ ALDH^bright^ cells had solely the wt-FLT3 protein present in two of the five cases tested. In 3/5 CD34-positive AML cases that we analyzed a very small amount of ALDH^bright^ progenitors contained FLT3-ITD and/or NPM1 mutations and might therefore be leukemic (Table S3B, Figure 5B).

### An ALDH Positive Population can be Identified in AML Patients

In AML, ALDH activity in leukemic blasts has been shown to define a subgroup with adverse prognosis and superior NOD/SCID engrafting potential [28], [29]. We focussed on differences in ALDH activity between HSC and LSC, whereby we identified a small population of high ALDH activity normal HSC (designated as ALDH^bright^). The ALDH^low^ compartment contained cells with various levels of ALDH activity within one AML patient but also inter-individual ALDH activity differences, relative to the ALDH^bright^ compartment. These differences were also seen in earlier studies [28], [33], [34] and we therefore compared our results with those. Seventeen AML samples (both CD34-positive and CD34-negative AML cases) were treated with DEAB in order to define an ALDH negative and positive population similarly as done by others [28], [29], [33](Figure S2 and Table S4). Similar to Cheung et al [28], we defined samples as ALDH-positive when the proportion of ALDH+ cells, defined by DEAB treatment, in the total sample was more than 5%. Three of our 17 cases (18%) were ALDH-positive (8.2%, 9.5%, 12.6%). In a study by Pearce et al, ALDH activity in AML was classified, based on the shape, level and scatter properties of the ALDH positive compartment, as rare, numerous or negative [33]. In our study 18/32 samples (56%) matched the ‘rare’ ALDH activity pattern and 14 matched the ‘numerous’ pattern (Figure S2A and S2B and Table S4).

## Discussion

In the present study, we have identified the activity of ALDH enzymes as a consistent functional discrimination between LSC and HSC concomitantly present in the BM of both CD34-negative and CD34-positive AML patients. Although HSC and LSC can, in a considerable part of AML cases, be distinguished using aberrancies of marker expression [12]–[14] and scatter properties [5], [16], assessment of ALDH activity enables such discrimination in all AML cases even in the absence of aberrancies. To our knowledge, there is no single parameter that allows identification of both normal and neoplastic stem cells in every AML patient. Association of high ALDH activity and ALDH1A1 expression solely with normal HSC and not with LSC within the AML BM may have important implications for treatment options, relapse prediction, and identification of HSC and LSC aiming at LSC-specific therapeutic target identification.

First, the relapse that occurs in 50% of AML patients after a seemingly successful chemotherapeutic treatment is likely caused by persistent LSC. This is reflected by the fact that frequency of LSC both at diagnosis and after treatment as well as expression of LSC signatures are tightly linked to the survival of AML patients [3], [5], [7]. For this reason, establishing LSC frequency at diagnosis and monitoring after treatment may become very important for clinical risk assessment and treatment decision making. Since cell surface markers are less stable due to interactions with the microenvironment or chemotherapy treatment, marker expression used nowadays for LSC detection might be less valuable than using a cytoplasmic functional maker like ALDH. In addition, heterogeneity of aberrant marker expression on LSC, i.e. either absent or present on part of the LSC compartment, and consequently the difficulty in identification of all LSC, will be circumvented by using ALDH activity as a marker.

Second, it is thought that LSC need to be eradicated in order to prevent relapse and to cure AML. The optimal anti-LSC therapy will spare the normal HSC. ALDH activity as a strong discriminative marker allows purification of LSC and HSC paving the way for LSC-specific target identification.

Third, it could be hypothesized that firm differences in ALDH activity between HSC and LSC in the BM of AML patients might result in consequent resistance of HSC for particular drugs subjected to ALDH-dependent detoxification. The high ALDH activity in HSC, compared to LSC, provided to be effective for LSC, predicts lower toxicity of alkylating agents, such as cyclophosphamide. The difference between the ALDH activity in LSC and HSC defines here the therapeutic window. We are currently testing drugs, known to be dependent on low ALDH activity for proper activity, i.e. LSC-specific killing. Besides this, we are using drug libraries to identify novel drugs that can be detoxified via high ALDH activity. We hypothesize that AML patients with a large difference in ALDH activity between HSC and LSC might benefit from ALDH activity dependent treatment strategies.

Lately, ALDH has received considerable attention as a functional marker for identification of cells with enhanced tumorigenic/metastatic potential and elevated therapeutic resistance in several cancers of epithelial origin [35]–[37]. The relative functional contribution of ALDH activity to tumor-initiating potential is not clear and has not been the subject of this study. It might be that leukemic cells with considerable higher ALDH activity than other leukemic cells have enrichment of leukemia-initiating potential or might be more resistant to therapy. As in the study of Gerber et al. [34], we observed various populations of LSC within the ALDH^low^ compartment (defined by the inhibition with DEAB) which might represent LSC populations with different levels of ALDH activity. In the study by Pearce et al [33], as well as in the study by Berger et al [34], ALDH+ cells with a normal genotype were found in part of AML cases. The low incidence of positive cases, as compared to the study of Pearce et al. [33], and the high number of ‘rare’ pattern cases, as compared to the study of Cheung et al. [28], that we found is possibly related to the high number of CD34-negative AML cases that we have included in our study.

The difference in ALDH activity level as seen between normal and neoplastic stem cells may also add to the discussion and controversies regarding the cell of origin for AML, i.e. derived from transformation of HSC or from more committed progenitors [38], [39]. The fact that AML LSC do not exhibit ALDH activity at levels as high as those of HSC suggests that the cell giving rise to AML LSC was a progenitor endowed only in part with stem cell features, with the exclusion of an enhanced ALDH activity.

Overall, we show a marked difference between ALDH activity of HSC and LSC within the AML BM indicating the importance of ALDH activity as a functional stem cell biomarker and its potential usefulness in identification and purification of HSC and LSC, with the aim of treatment decision making, relapse prediction and development of LSC-specific therapies.

## Methods

### Patient Samples, AML Blast Phenotype, and Purification

BM samples were collected after informed consent from 32 AML patients at diagnosis. Normal BM was obtained after informed consent from healthy donors or patients undergoing cardiac surgery. The patients we used in this study gave informed consent following the procedure that was approved by the Medical Ethical Committee of our institute, the METC-VUmc. We (the Hematology department of the VUMC) have formal approval from the METC-VUmc for all our studies in acute myeloid leukemia patients. The patients gave written informed consent and the material of patients that did not gave this consent is not used for research purposes. AML phenotyping, cytogenetic, and molecular genetic analysis were carried out in the context of routine diagnostics at our facility. Cells were analyzed freshly or after thawing of samples frozen in liquid nitrogen. Mononuclear cells were isolated by Ficoll gradient (1.077 g/ml Amersham Biosciences, Freiburg Germany). Red blood cells were lysed by 10 minutes incubation on ice, using 10 ml lysing solution containing 155 mM NH4Cl, 10 mM KHCO3, 0.1 mM Na2EDTA pH7.4 applied directly to the cell pellet. AML cells were frozen in RPMI (Gibco, Paisley, Uk) with 20% heat-inactivated fetal bovine serum (FBS, Greiner, Alphen a/d Rijn, The Netherlands) and 10% dimethylsulfoxide (Riedel-de Haen, Seelze, Germany) in isopropanol-filled containers and subsequently stored in liquid nitrogen. When needed for analysis, cells were thawed and suspended in pre-warmed RPMI with 40% FBS at 37°C. Cells were washed in PBS with 0.1% HSA.

### Aldehyde Dehydrogenase Activity Assay

Primary AML cells (1×10^6^/ml) were resuspended in the aldefluor assay buffer at a concentration of 2×10^6^ cells/ml. Cells were incubated with the ALDH-substrate BAAA (Stem cell Technologies, Aldefluor assay, 1 µl/ml) with or without the ALDH inhibitor, diethylamino-benzaldehyde (DEAB, 1.5 mM in 95% ethanol) and incubated at 37°C for 60 minutes according to the manufacturer protocol. After performing the Aldefluor assay, cells were resuspended in icecold PBS/0.1%HSA and washed once. The BAAA substrate can be detected in the FITC channel of the flow cytometer.

### Cell Labeling, Flow Cytometry and Cell Purification

AML cells were incubated for 30 minutes on ice with monoclonal antibody (MoAb) combinations consisting of phycoerythrin (PE), allophycocyanin (APC), R-phycoerythrin-cyanine 7 (PC7), peridinin chlorophyll protein (PERCP) labeled MoAbs, anti-CD45 PerCp, Anti-CD34 PC7, Anti-CD38 APC, and anti-CD7 PE, Anti-CD56 PE, Anti-CLL1 PE, Anti-CD19 PE, Anti-CD33 PE, Anti-CD22 PE, Anti-CD123 PE. The antibodies were all from BD Biosciences (BD Biosciences, San Jose, CA, USA). After antibody staining, cells were washed with icecold PBS/0.1%HSA, resuspended in 250 µl cold PBS/0.1%HSA and stained for 5 minutes with Sytox Blue (Invitrogen, Molecular Probes) enabling to exclude dead cells. Cells were kept on ice before FACS analysis. Labeled samples were analyzed and cells were sorted using a flow cytometer (BD FACSAria, equipped with red, blue, ultra-violet and infrared solid-state lasers; BD Biosciences, San Jose, CA, USA). Prior to molecular analysis, subfractions were sorted. Data acquisition was performed using either a FACS Calibur or a FACS CantoII, both from BD Biosciences. Analysis was performed using Cellquest and FACS Diva software (BD Biosciences). For analysis of the presence molecular aberrancies, cells were sorted directed into cold culture medium. Purity of sorted population was in most cases >98%.

### Length Fragment Analysis of FLT3 and NPM1 Mutations

To determine mutational status of flow cytometer-sorted subpopulations, only cryo-preserved cells were used. Genomic DNA (gDNA) was isolated from pellets of cells according to manufacturer’s instructions using the Quiagen Allprep kit (Quiagen Benelux B.V., Venlo, The Netherlands). Mutation profiling was performed on isolated gDNA as previously described for *FLT3/ITD and NPM1.* Direct PCR was applied to cell sorted subfractions under similar reaction conditions, except for the use of lower reaction volumes (always 10 µl). Mutations in NPM1exon12 were analyzed with PCR with the following primers: NPM1forward: 5′-TTAACTCTCTGGTGGTAGAATGA-3′; NPM1 reverse: 5-CTGACCACCGCTACTATGT-3, located in intron 11 and exon12 of the NPM1 genomic DNA, respectively. FLT3 was amplified using the primers spanning the entire transmembrane and JM domains of FLT3. Subsequent fragment analysis was performed with a tetrachlorofluorescein phosphoramidite-labeled forward primer (Biolegio, Nijmegen, The Netherlands). For both FLT3 and NPM1 analysis, lymphocytes served as an internal negative control.

### RNA Sequencing

Total RNA from cells (normal BM HSC, AML HSC and LSC) was extracted with TRIzol reagent (Invitrogen, Leek, The Netherlands) according to the manufacturer’s protocol. The quality of RNA samples was measured on a 2100 Bioanalyzer (Agilent, Amstelveen, Netherlands). The sequencing libraries, each with individual Illumina indexes, were constructed using the TruSeq™ RNA Sample Prep Kit v2 (Illumina, San Diego, CA, USA) according to the manufacturer’s instructions. The resulting library DNA concentration and molarity was checked on a 2100 Bioanalyzer. Next, a mixture of three 10 pM libraries were pooled equimolar and the resulting DNA was clustered onto a V3 flowcell lane using a c-Bot cluster station and subsequently sequenced in single read fashion for 50 bp using the Illumina HiSeq2000 and V3 SBS Chemistry. Sequence reads were aligned to the human reference genome. In order to determine whether the aldehyde dehydrogenases are differentially expressed between samples, the R package DEGseq was used [40].

## Supporting Information

Figure S1 **CD34-positive AML cases; variation in the difference in ALDH activity between ALDH^bright^ and ALDH^low^ compartments.** ALDH versus SCC of the CD34+CD38– compartment of three CD34-positive AML cases. ALDH activity segregates the CD34+CD38– compartment in CD34+CD38– ALDH^bright^ and CD34+CD38– ALDH^low^ cells. One AML case, AML-1048, with a relatively small difference between HSC and LSC ALDH activity levels (panel 1). An AML case, AML-1036, with a large difference between ALDH activity levels of HSC and LSC (panel 2). One AML case, AML-1013, with mainly HSC (panel 3).
(TIF)

Figure S2 **Defining the ALDH compartments as has been done by others, amount of ALDH positive cells and pattern (29,34).** The amount of ALDH positive cells is determined (normalized with the DEAB inhibitor) and the samples are defined as ALDH- (<5% ALDH positive cells from the total AML, **A**) or ALDH+ (>5% ALDH positive cells, **B**) With this method used by Cheung et al. (28) AML patients are classified based on percentage of ALDH positive cells defined by DEAB treatment. Our classification shows that 18% of AML patients are positive for ALDH (more than 5% of cells are ALDH+). The pattern of ALDH activity is determined by the shape, level and scatters properties of the ALDH activity as defined by Pearce et al. (33) The “rare” pattern ALDH activity is seen in (**A**) and the numerous pattern of ALDH activity is seen in (**B**). All the positive AML cases have the numerous pattern.
(TIF)

Table S1 **Quantification of ALDH activity in marker defined CD34+CD38**– **HSC and CD34+CD38**– **LSC compartments in CD34 positive AML.** MFI values of CD34+CD38–marker- and CD34+CD38–marker+ were standardized by dividing them with the ALDH-MFI values from lymphocytes present in the same sample. AML samples were treated with diethylamino-benaldehyde (DEAB) to compare background MFI values in each cell population. Comparison of the median of the ALDH-MFI values of two populations; CD34+CD38– marker- cells and CD34+CD38– marker+ cells from 9 (9/19 CD34-positive AML cases were treated with DEAB) CD34-positive AML samples (median values are indicated) was done. In all CD34-positive AML cases, the MFI of CD34+CD38–marker- cells was divided with the MFI of the CD34+CD38–marker+ cells to obtain fold induction of ALDH activity in HSC compared to LSC within the AML. MFI is mean fluorescent intensity.
(TIF)

Table S2 **Quantification of ALDH activity in marker defined CD34+CD38**– **HSC and CD34**– **compartments in CD34 negative AML cases.** *MFI is mean fluorescent intensity. MFI values of CD34+CD38–marker- cells and CD34– cells were standardized by dividing them with the ALDH-MFI values from lymphocytes present in the same sample. AML samples were treated with diethylamino-benaldehyde (DEAB) to compare background MFI values in each cell population. Comparison of the median of the ALDH-MFI values of two populations; CD34+CD38– marker- cells and CD34– cells from 8 (8/14 CD34-negative AML cases were treated with DEAB) CD34-negative AML samples (median values are indicated) was done. In all CD34-negative AML cases, the MFI of CD34+CD38–marker- cells was divided by the MFI of the CD34– cells to obtain fold induction of ALDH activity in HSC compared to the bulk of the AML. *MFI is mean fluorescent intensity.
(TIF)

Table S3 **The presence of FLT3-ITD and mutated NPM1 in ALDH^bright^ CD34+CD38dim/+ progenitor populations.** (**A**) CD34-negative AML cases. 4/4 CD34+CD38+/dim cells were negative. (**B**) CD34-positive AML cases. # In 3/5 CD34-positive AML cases, the CD34+CD38+ progenitor population has a tiny population of mutated cells present (likely contamination with ALDH^low^ cells). ne: not evaluable because CLL-1 (and sometimes CD33) is not a reliable aberrant marker for malignancy of CD34+CD38+ progenitors since part of normal progenitors and more mature CD34– cells can have CLL-1 and lineage marker expression (12). na: not applicable because no leukemia-associated aberrant marker present or expression to low.
(TIF)

Table S4 **Classification of ALDH defined AML cases.** Percentage of ALDH positivity defined by DEAB treatment in the total AML (Panel 2). Percentage of ALDH positivity defined by DEAB treatment in the CD45dim blast population of cells (panel 3). AML cases that are defined as positive (more than 5% of the total AML population) are indicated as plus. AML cases that are defined as negative (less than 5% of the total AML population) are indicated as min (panel 4, Cheung et al.^28^). AML cases were defined as rare or numerous (panel 4, Pearce et al.^33^). In our cohort there are no AML cases with the negative pattern. The percentage of CD45dim blasts in the total AML (panel 6), the frequency of CD34+ cells within the total CD45dim population (panel 7), the total amount of CD34+CD38– cells within the CD34+ compartment (panel 8) and the total amount of CD34+CD38– cells within the CD45dim population of cells (panel 9) is indicated in this table.
(TIF)
