# Supplementary figures and images for: Normal Hematopoietic Stem Cells within the AML Bone Marrow Have a Distinct and Higher ALDH Activity Level than Co-Existing Leukemic Stem Cells
Source: PLoS One. 2013 Nov 11;8(11):e78897. doi: 10.1371/journal.pone.0078897 (PMC3823975; doi:10.1371/journal.pone.0078897)

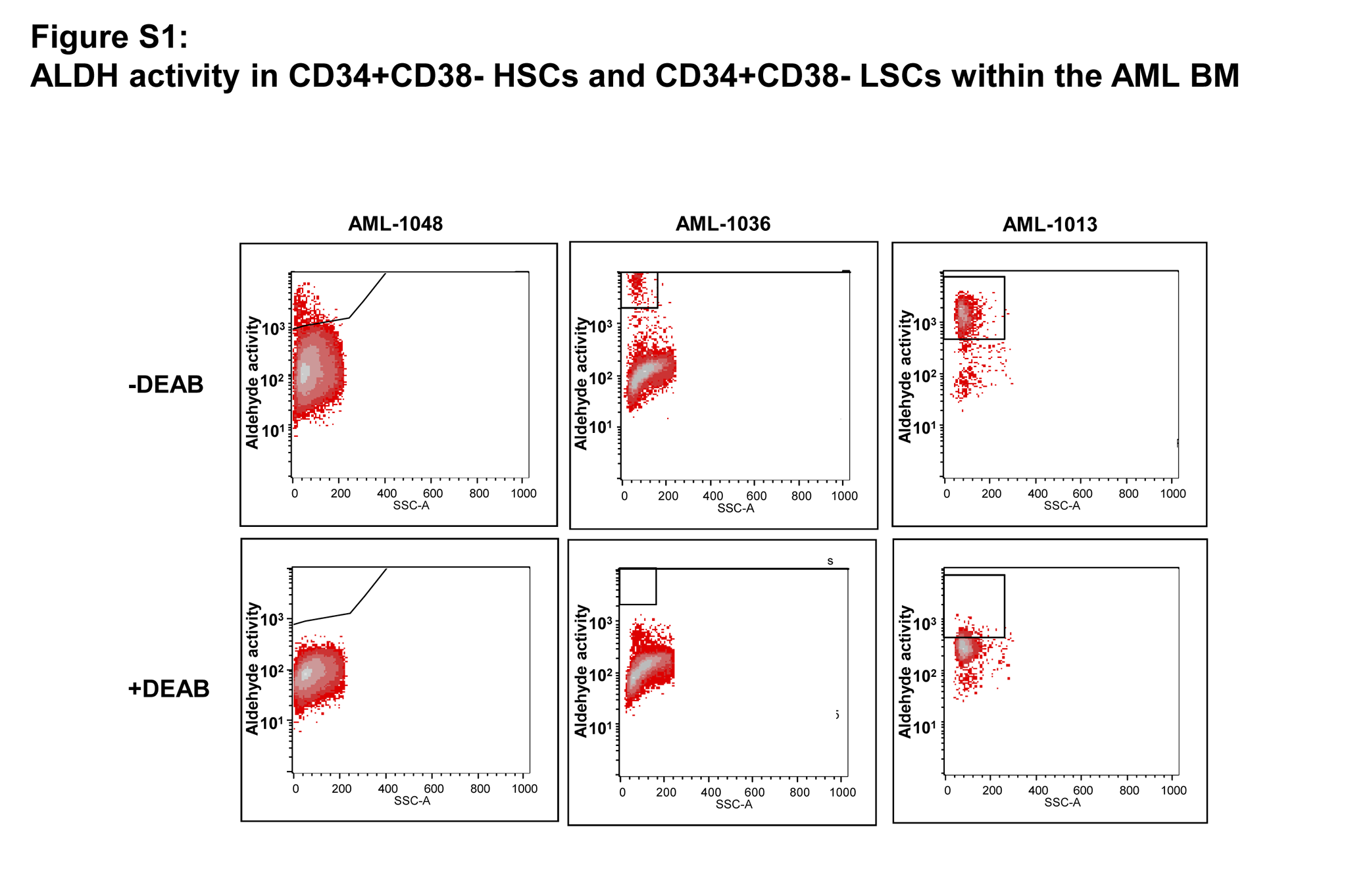

Supplement: Figure S1 — CD34-positive AML cases; variation in the difference in ALDH activity between ALDHbright and ALDHlow compartments. ALDH versus SCC of the CD34+CD38– compartment of three CD34-positive AML cases. ALDH activity segregates the CD34+CD38– compartment in CD34+CD38– ALDHbright and CD34+CD38– ALDHlow cells. One AML case, AML-1048, with a relatively small difference between HSC and LSC ALDH activity levels (panel 1). An AML case, AML-1036, with a large difference between ALDH activity levels of HSC and LSC (panel 2). One AML case, AML-1013, with mainly HSC (panel 3). (TIF) [file pone.0078897.s001.tif]

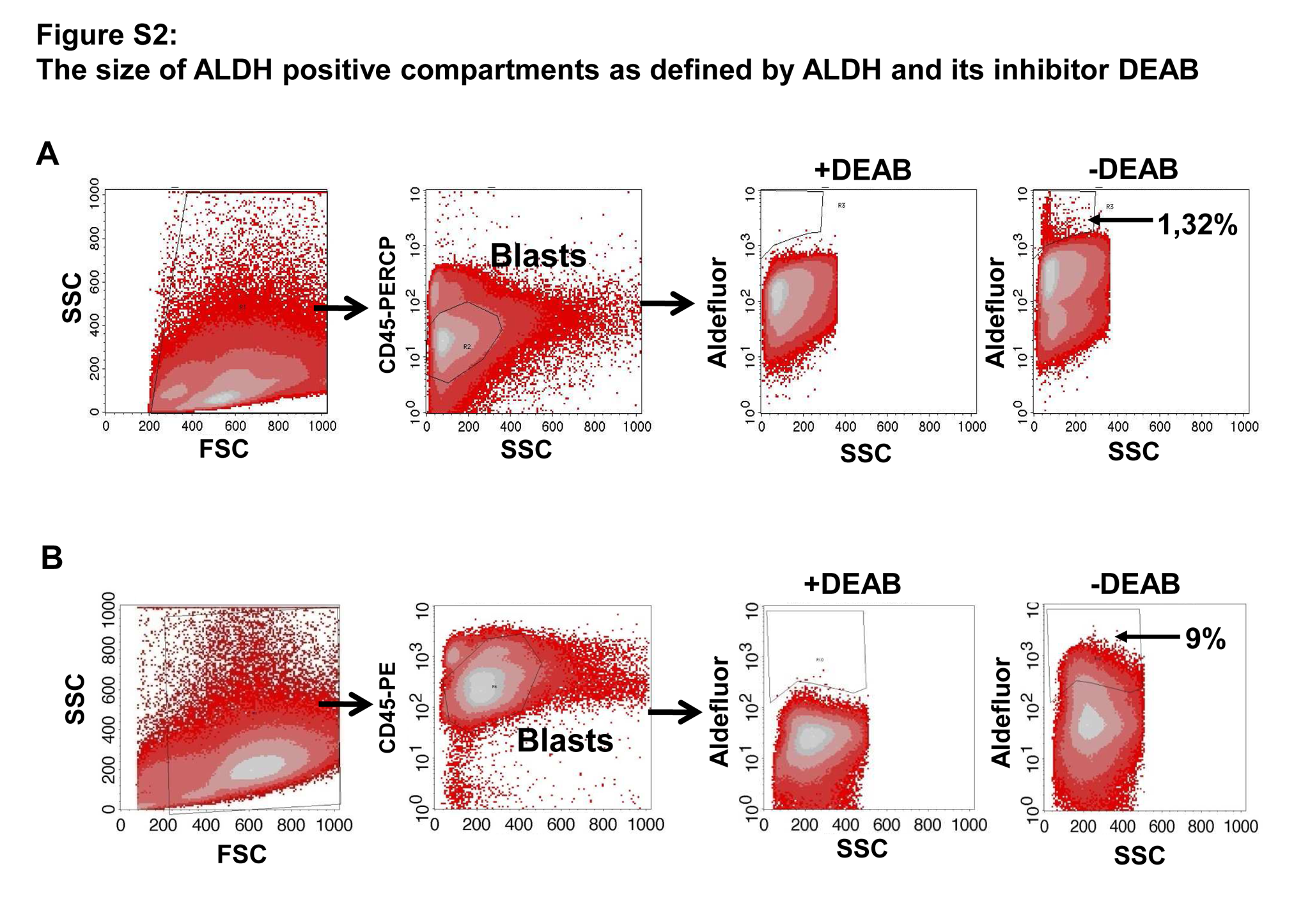

Supplement: Figure S2 — Defining the ALDH compartments as has been done by others, amount of ALDH positive cells and pattern (29,34). The amount of ALDH positive cells is determined (normalized with the DEAB inhibitor) and the samples are defined as ALDH- (<5% ALDH positive cells from the total AML, A) or ALDH+ (>5% ALDH positive cells, B) With this method used by Cheung et al. (28) AML patients are classified based on percentage of ALDH positive cells defined by DEAB treatment. Our classification shows that 18% of AML patients are positive for ALDH (more than 5% of cells are ALDH+). The pattern of ALDH activity is determined by the shape, level and scatters properties of the ALDH activity as defined by Pearce et al. (33) The “rare” pattern ALDH activity is seen in (A) and the numerous pattern of ALDH activity is seen in (B). All the positive AML cases have the numerous pattern. (TIF) [file pone.0078897.s002.tif]

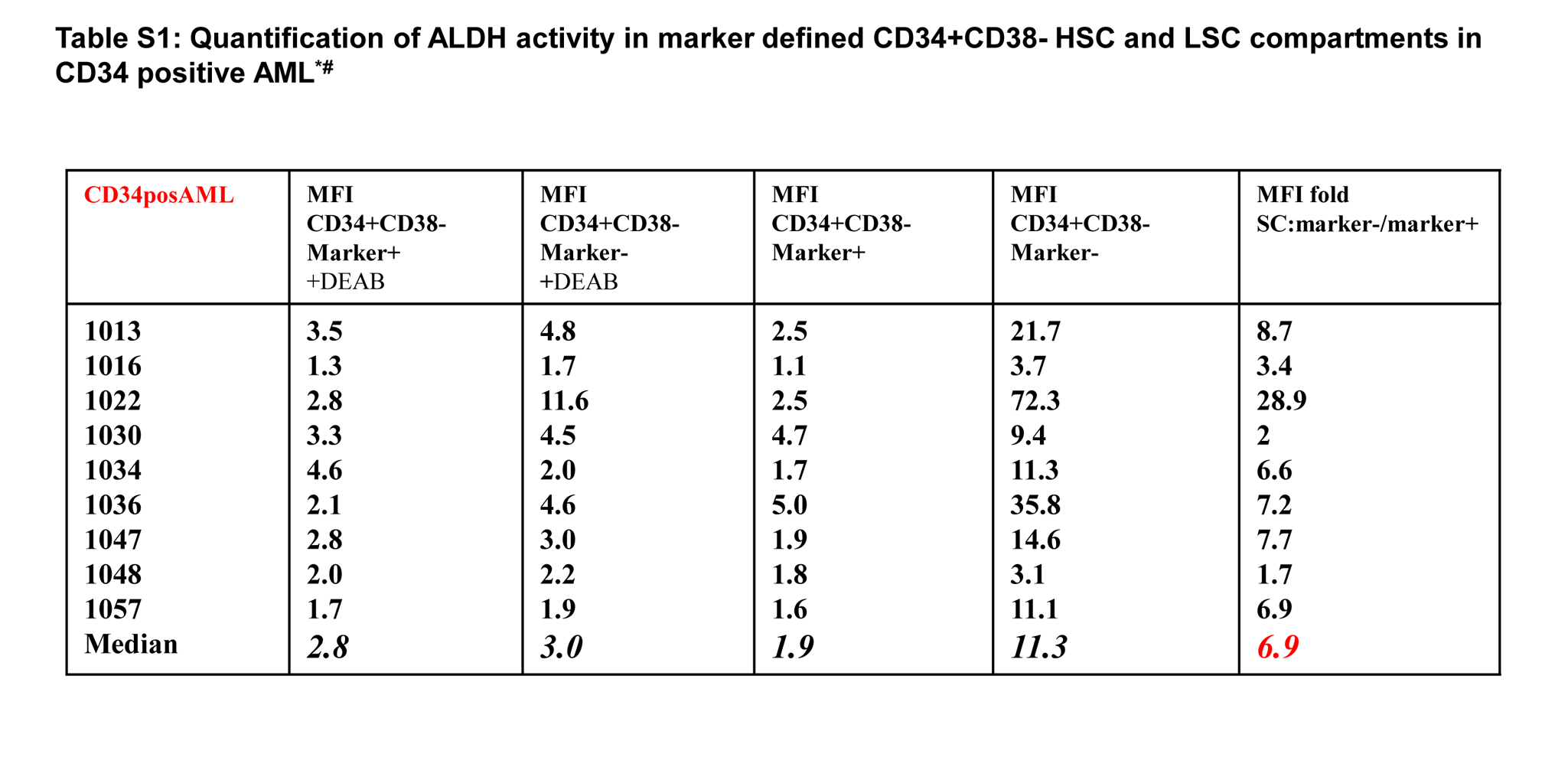

Supplement: Table S1 — Quantification of ALDH activity in marker defined CD34+CD38– HSC and CD34+CD38– LSC compartments in CD34 positive AML. MFI values of CD34+CD38–marker- and CD34+CD38–marker+ were standardized by dividing them with the ALDH-MFI values from lymphocytes present in the same sample. AML samples were treated with diethylamino-benaldehyde (DEAB) to compare background MFI values in each cell population. Comparison of the median of the ALDH-MFI values of two populations; CD34+CD38– marker- cells and CD34+CD38– marker+ cells from 9 (9/19 CD34-positive AML cases were treated with DEAB) CD34-positive AML samples (median values are indicated) was done. In all CD34-positive AML cases, the MFI of CD34+CD38–marker- cells was divided with the MFI of the CD34+CD38–marker+ cells to obtain fold induction of ALDH activity in HSC compared to LSC within the AML. MFI is mean fluorescent intensity. (TIF) [file pone.0078897.s003.tif]

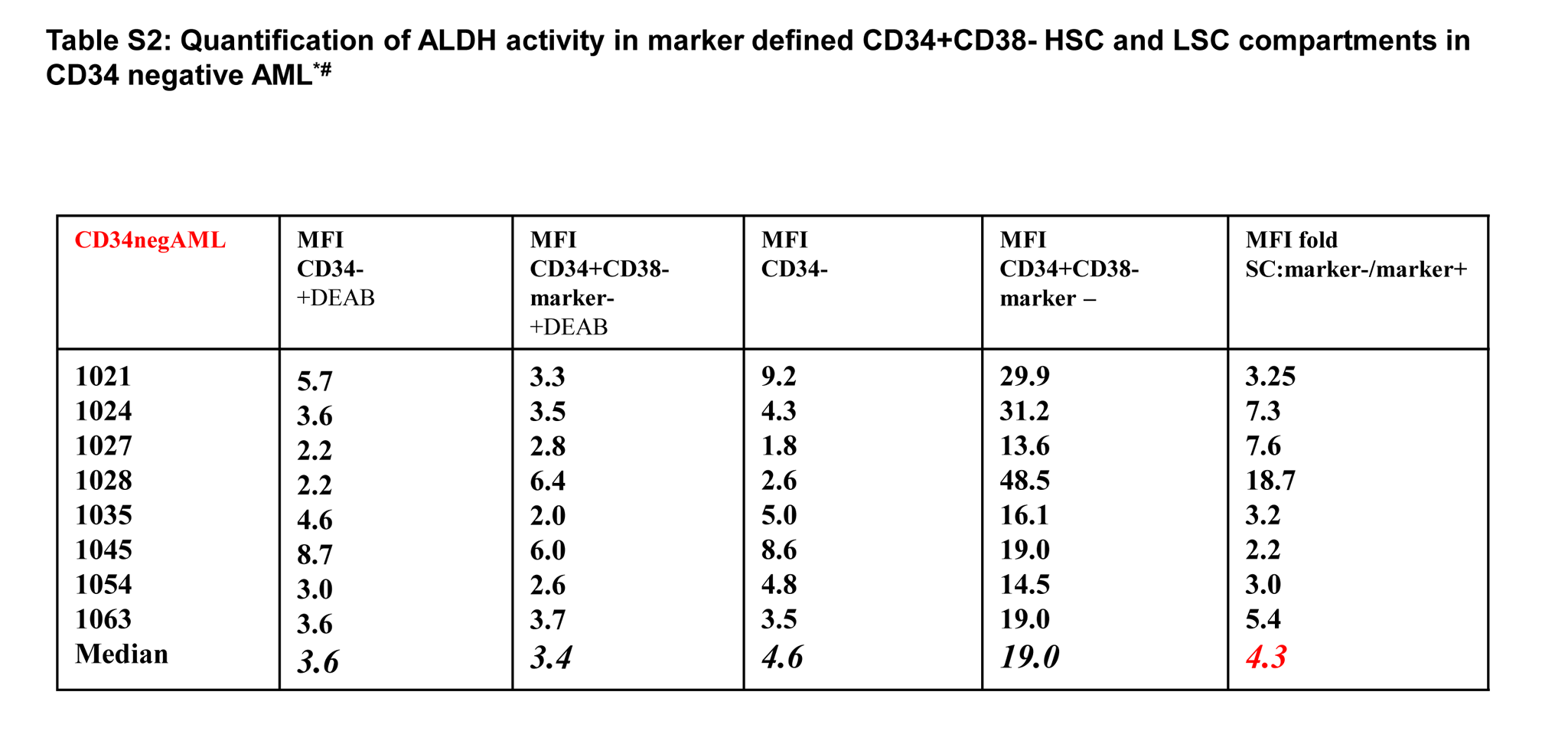

Supplement: Table S2 — Quantification of ALDH activity in marker defined CD34+CD38– HSC and CD34– compartments in CD34 negative AML cases. *MFI is mean fluorescent intensity. MFI values of CD34+CD38–marker- cells and CD34– cells were standardized by dividing them with the ALDH-MFI values from lymphocytes present in the same sample. AML samples were treated with diethylamino-benaldehyde (DEAB) to compare background MFI values in each cell population. Comparison of the median of the ALDH-MFI values of two populations; CD34+CD38– marker- cells and CD34– cells from 8 (8/14 CD34-negative AML cases were treated with DEAB) CD34-negative AML samples (median values are indicated) was done. In all CD34-negative AML cases, the MFI of CD34+CD38–marker- cells was divided by the MFI of the CD34– cells to obtain fold induction of ALDH activity in HSC compared to the bulk of the AML. *MFI is mean fluorescent intensity. (TIF) [file pone.0078897.s004.tif]

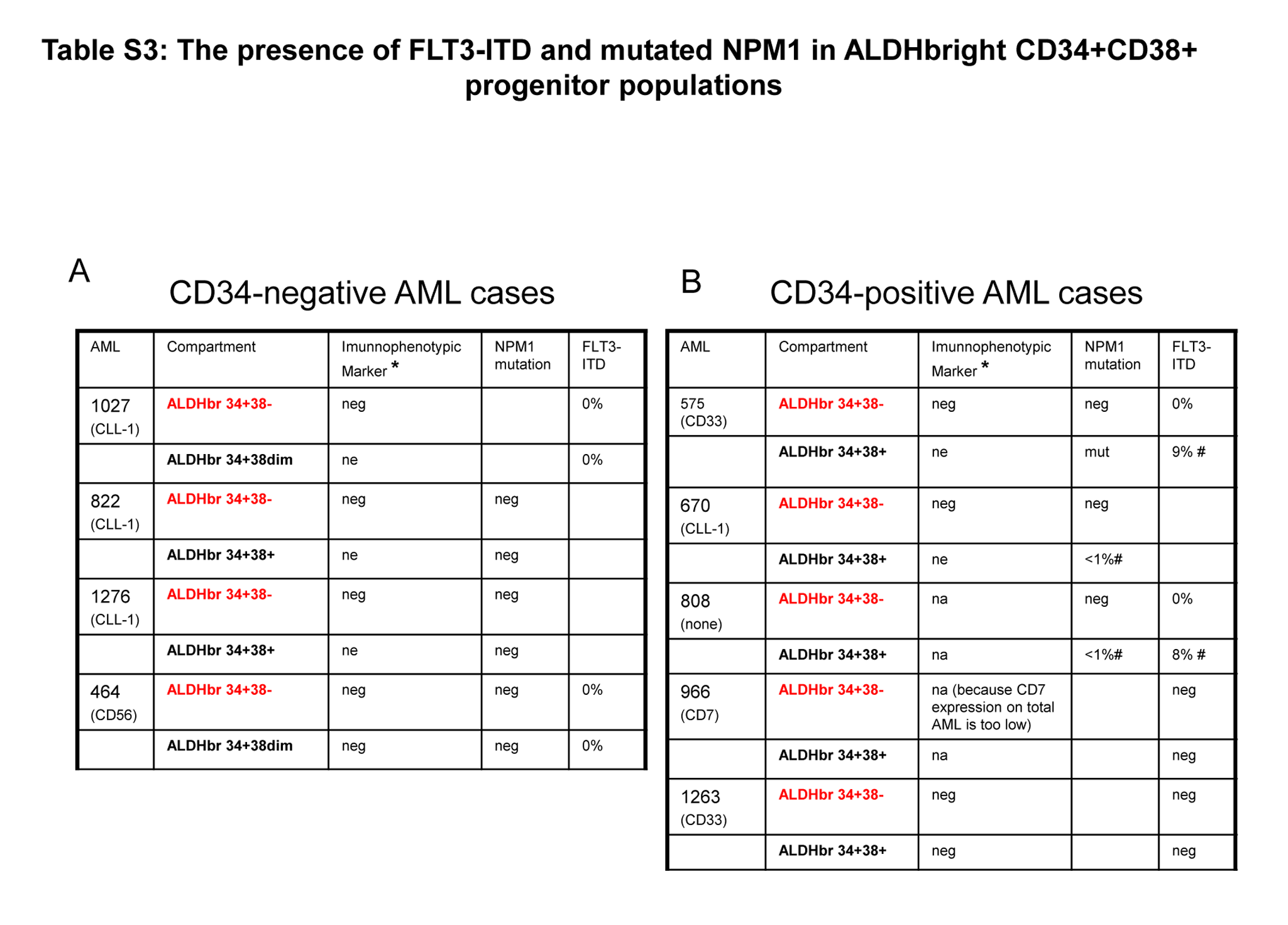

Supplement: Table S3 — The presence of FLT3-ITD and mutated NPM1 in ALDHbright CD34+CD38dim/+ progenitor populations. (A) CD34-negative AML cases. 4/4 CD34+CD38+/dim cells were negative. (B) CD34-positive AML cases. # In 3/5 CD34-positive AML cases, the CD34+CD38+ progenitor population has a tiny population of mutated cells present (likely contamination with ALDHlow cells). ne: not evaluable because CLL-1 (and sometimes CD33) is not a reliable aberrant marker for malignancy of CD34+CD38+ progenitors since part of normal progenitors and more mature CD34– cells can have CLL-1 and lineage marker expression (12). na: not applicable because no leukemia-associated aberrant marker present or expression to low. (TIF) [file pone.0078897.s005.tif]

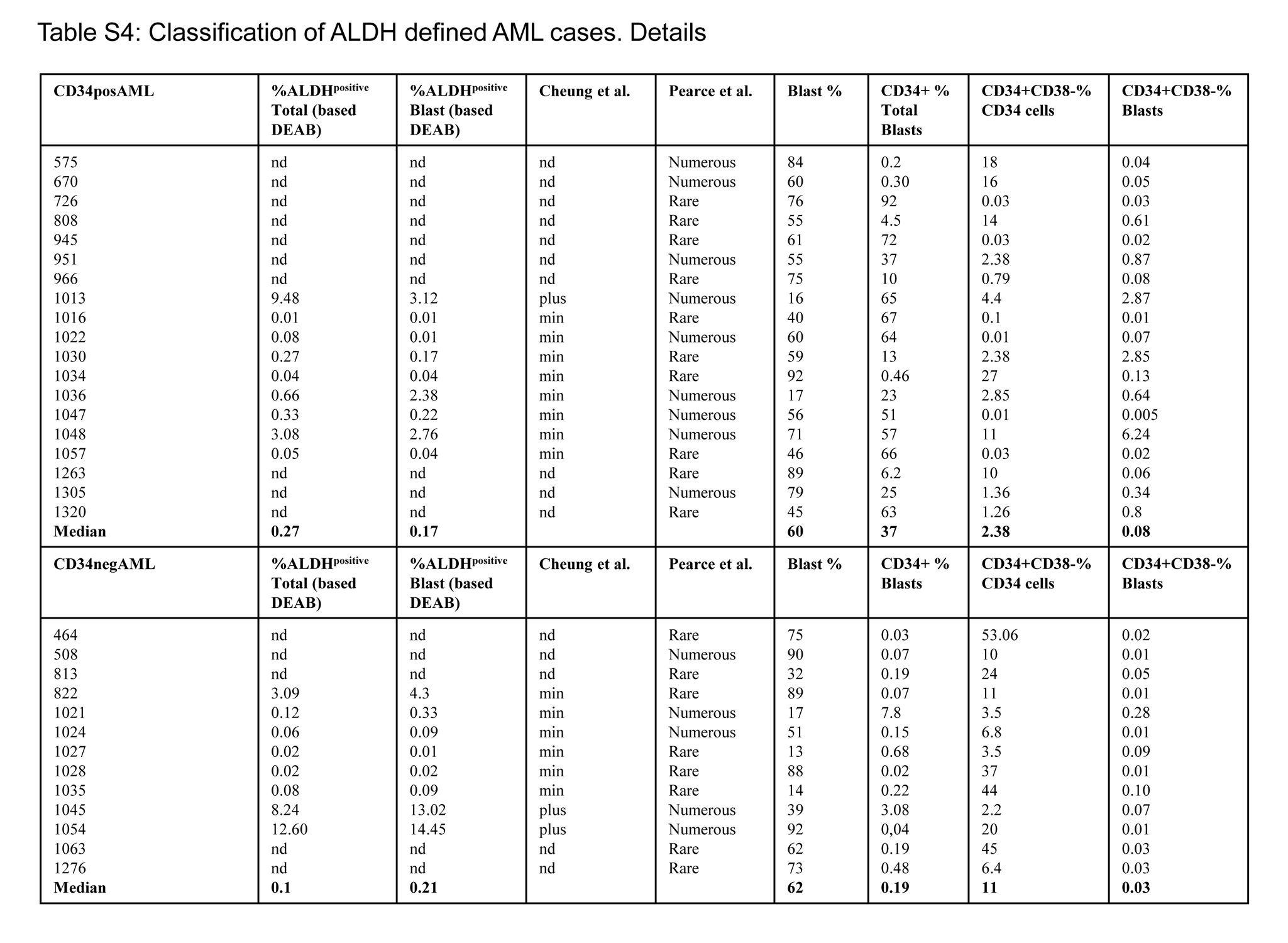

Supplement: Table S4 — Classification of ALDH defined AML cases. Percentage of ALDH positivity defined by DEAB treatment in the total AML (Panel 2). Percentage of ALDH positivity defined by DEAB treatment in the CD45dim blast population of cells (panel 3). AML cases that are defined as positive (more than 5% of the total AML population) are indicated as plus. AML cases that are defined as negative (less than 5% of the total AML population) are indicated as min (panel 4, Cheung et al.28). AML cases were defined as rare or numerous (panel 4, Pearce et al.33). In our cohort there are no AML cases with the negative pattern. The percentage of CD45dim blasts in the total AML (panel 6), the frequency of CD34+ cells within the total CD45dim population (panel 7), the total amount of CD34+CD38– cells within the CD34+ compartment (panel 8) and the total amount of CD34+CD38– cells within the CD45dim population of cells (panel 9) is indicated in this table. (TIF) [file pone.0078897.s006.tif]
